# A196 CSF2 AUTOANTIBODIES AS A SEROLOGICAL MARKER FOR CROHN'S DISEASE

**DOI:** 10.1093/jcag/gwad061.196

**Published:** 2024-02-14

**Authors:** S Tai, D Del Valle, R Urango, K Croitoru, S Gnjatic, J Korzenik, J Colombel, A Mortha

**Affiliations:** Immunology, University of Toronto, Toronto, ON, Canada; Icahn School of Medicine at Mount Sinai Tisch Cancer Institute, New York, NY; Icahn School of Medicine at Mount Sinai, New York, NY; Immunology, University of Toronto, Toronto, ON, Canada; Icahn School of Medicine at Mount Sinai Tisch Cancer Institute, New York, NY; Brigham and Women's Hospital, Boston, MA; Icahn School of Medicine at Mount Sinai Department of Genetics and Genomic Sciences, New York, NY; Immunology, University of Toronto, Toronto, ON, Canada

## Abstract

**Background:**

Crohn’s disease (CD) is a chronic inflammatory disorder of the gastrointestinal tract that severely affects quality of life. Despite technological advances, CD diagnoses remain difficult and invasive, with delayed diagnoses correlated with worse prognosis and complications. CD is multifactorial, involving complex interactions between genetic and environmental risk factors, which leads to difficulty in identifying a cause and cure. However, research alludes to an underlying dysregulation of immune activity that leads to chronic intestinal inflammation. Mononuclear phagocytes (MNP) are essential immune cells that promote intestinal homeostasis through supporting immune tolerance, antimicrobial activity, and barrier integrity. A crucial cytokine that promotes the survival and function of intestinal MNP is Colony Stimulating Factor 2 (CSF2). Intriguingly, previous work in our lab demonstrated that CSF2 autoantibodies (CSF2-Ab) can be detected in the serum of every third CD patient and antedate the onset of CD by up to 6 years. In contrast, these titers are not seen in healthy donors or ulcerative colitis. Moreover, CSF2-Ab were predictive of disease location and severity, and are able to neutralize CSF2 by binding to glycosylations, impacting downstream signaling in MNP. These data suggest a role for CSF2-Ab in promoting intestinal immune dysregulation in CD.

**Aims:**

Our work aims to characterize CSF2-Ab and their role in CD.

**Methods:**

We developed a bead-based flow cytometric assay to screen CD patient serum for CSF2-Ab in several cohorts that contain samples at time points prior to, at, and after diagnosis.

**Results:**

Our preliminary data demonstrate that our assay is rapid and specific for detecting CSF2-Ab of various isotypes in human serum and can be validated using ELISA. Furthermore, we show that this assay can be used to determine the epitope specificity of CSF2-Ab in CD patients in just one single sample.

**Conclusions:**

We have developed a rapid and accessible assay to predict CD development years before diagnosis using minimal serum samples. Moreover, this screen will enable subclassification of patients based on their autoantibody reactivity to glycovariants of CSF2. Glycovariants that escape recognition by CD-specific CSF2-Ab could potentially be used as a therapeutic to ameliorate disease. Beyond treatment, understanding how CSF2-Ab epitope specificity and isotypes may change over the course of disease development will serve as a roadmap for elucidating the role of CSF2-Ab in CD.

**Funding Agencies:**

CAG, CIHR

